# Potential important roles and signaling mechanisms of YPEL4 in pulmonary diseases

**DOI:** 10.1186/s40169-018-0194-5

**Published:** 2018-06-11

**Authors:** Lillian Truong, Yun-Min Zheng, Tengyao Song, Yi Tang, Yong-Xiao Wang

**Affiliations:** 1 Department of Molecular and Cellular Physiology, Albany Medical College, 47 New Scotland Avenue (MC8), Albany, NY 12208 USA; 2 Department of Regenerative and Cancer Cell Biology, Albany Medical College, Albany, NY 12208 USA

**Keywords:** Cell cycle, Centrosome, Proliferation, Pulmonary disease, Yippee-like-4

## Abstract

**Background:**

Human Yippee-like-4 (YPEL4) is a member of the YPEL gene family. This family has been characterized as the first highly conserved family of genes coding for proteins that contain putative zinc-finger-like metal-binding domains, known as the Yippee domain. The YPEL family proteins are located at the centrosome adjacent to the nucleolus during interphase and mitotic apparatus during mitosis. Due to its subcellular localization, it is believed that YPEL4 may have an important role in the cell cycle and proliferation. Recent studies have shown the involvement of YPEL4 in biological processes such as the mitogen-activated protein kinase pathway and adrenal cell proliferation. Research on YPEL4 up to date also suggests that YPEL4 is a very important player in pulmonary diseases.

**Conclusions:**

YPEL4 may regulate the mitogen-activated protein kinase signaling pathway to mediate adrenal cell proliferation; this molecule is also likely to be a very important player in pulmonary diseases. Although the function of YPEL4 is largely unknown, further research may substantiate the functional importance and underlying molecular processes in pulmonary and other diseases that would allow YPEL4 to become a therapeutic target.

## Introduction

The *YPEL* gene family consists of members *YPEL1* through *YPEL5*, all of which have high homology with *Drosophila yippee* gene. Characterization of the gene family showed a family of protein-coding genes containing putative zinc-finger-like metal-binding domains, also known as the Yippee domain, with molecular masses ranging from 13.5 to 17.5 KD. In search for YPEL family genes from public databases such as DNA Data Bank of Japan (DDBJ), European Molecular Biological Laboratory (EMBL), and GenBank, 100 genes from 68 species were found including animals, plants and fungi, suggesting that the gene family is conserved in a wide range of eukaryotic species [1]. The amino acid sequences of the 100 YPEL proteins were similar and without exception, a consensus sequence of C-X_2_-C-X_19_-G-X_3_-L-X_5_-N-X_13_-G-X_8_-C-X_2_-C-X_4_-GWXY-X_10_-K-X_6_-E was established for all the YPEL family proteins. These proteins are located at the centrosome, adjacent to the nucleolus and mitotic apparatus, and are universally expressed in all eukaryotes [1].

**Fig. 1:**
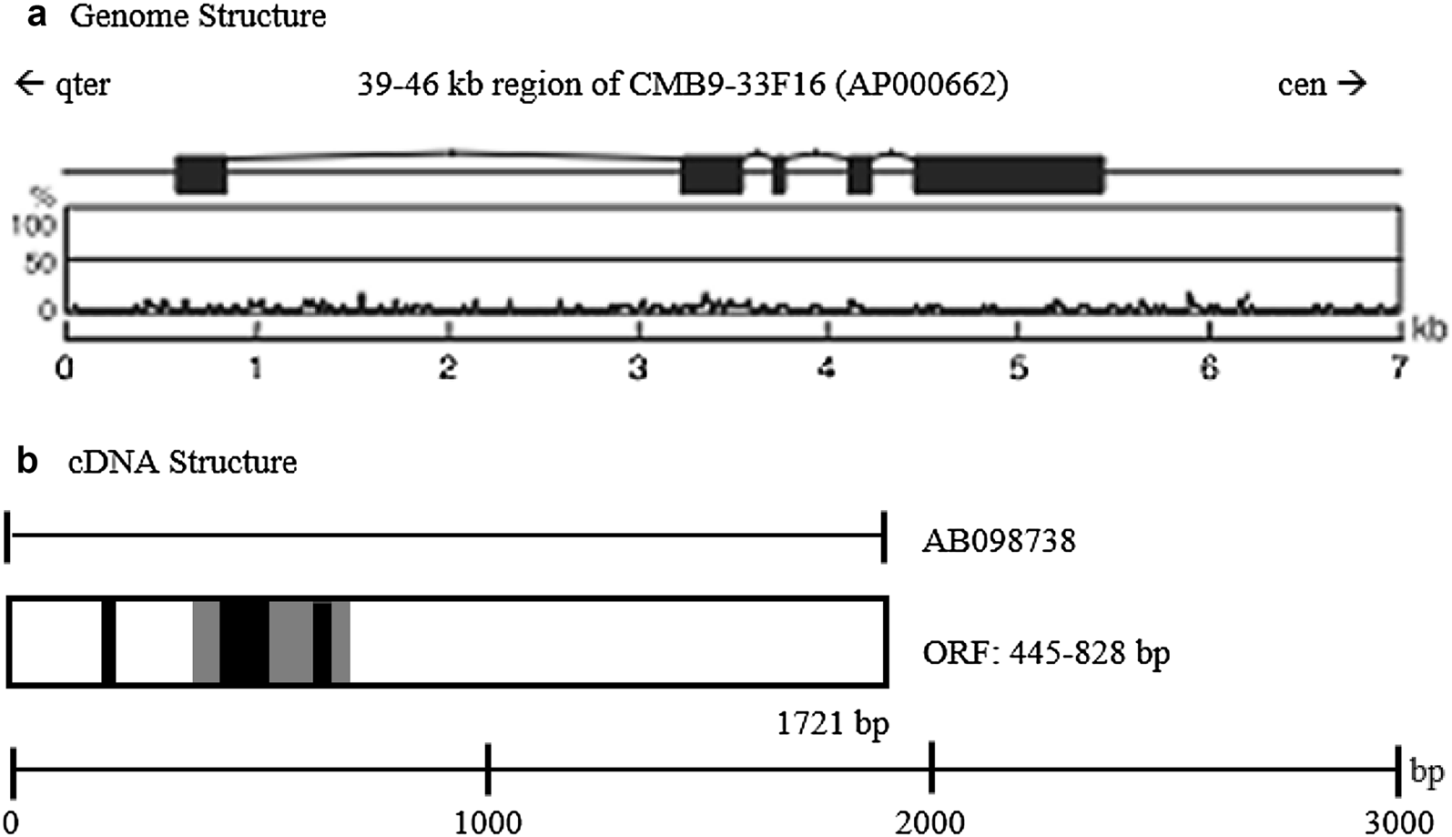
The genomic and cDNA structures of the human *YPEL4* gene (YPEL4 11q12.1). **a** Exon/intron organization and coding/non-coding regions on cDNA. Solid lines show CpG frequency. **b** cDNA was drawn as left side for 5′ end. The gray area on cDNA represents the ORF. Positions of various PCR products were indicated on cDNA [1]

Human *YPEL4*, or Yippee-Like 4, is a member of the *YPEL* gene family, which is located at 11q12.1. The genomic and cDNA structures of the human *YEL4* gene are shown in Fig. 1. Ensembl analysis of obtained mouse *Ypel4* cDNA sequences revealed the chromosomal localization and exon number of these genes, which could then be compared to the human orthologue *YPEL4*. In both mice and humans *YPEL4*, there are 5 exons and the open reading frame (ORF) contains 384 base pairs (bp) [1]. As described above, the genomic location of the human *YPEL4* gene is 11q12.1, whereas the genomic location of the mouse Ypel4 gene is 2qE1 [1]. On chromosome 11, the start of the human *YPEL4* gene begins at the 57,645,087 bp from the Phosphotriesterase Related gene (PTER) and ends at the 57,649,944 bp from PTER, a total of 4858 bases (Gene Cards [2]). Human *YPEL4* has 10 splicing variants, 63 orthologs, and 4 paralogs [3].

## YPEL4 expression

**Fig. 2:**
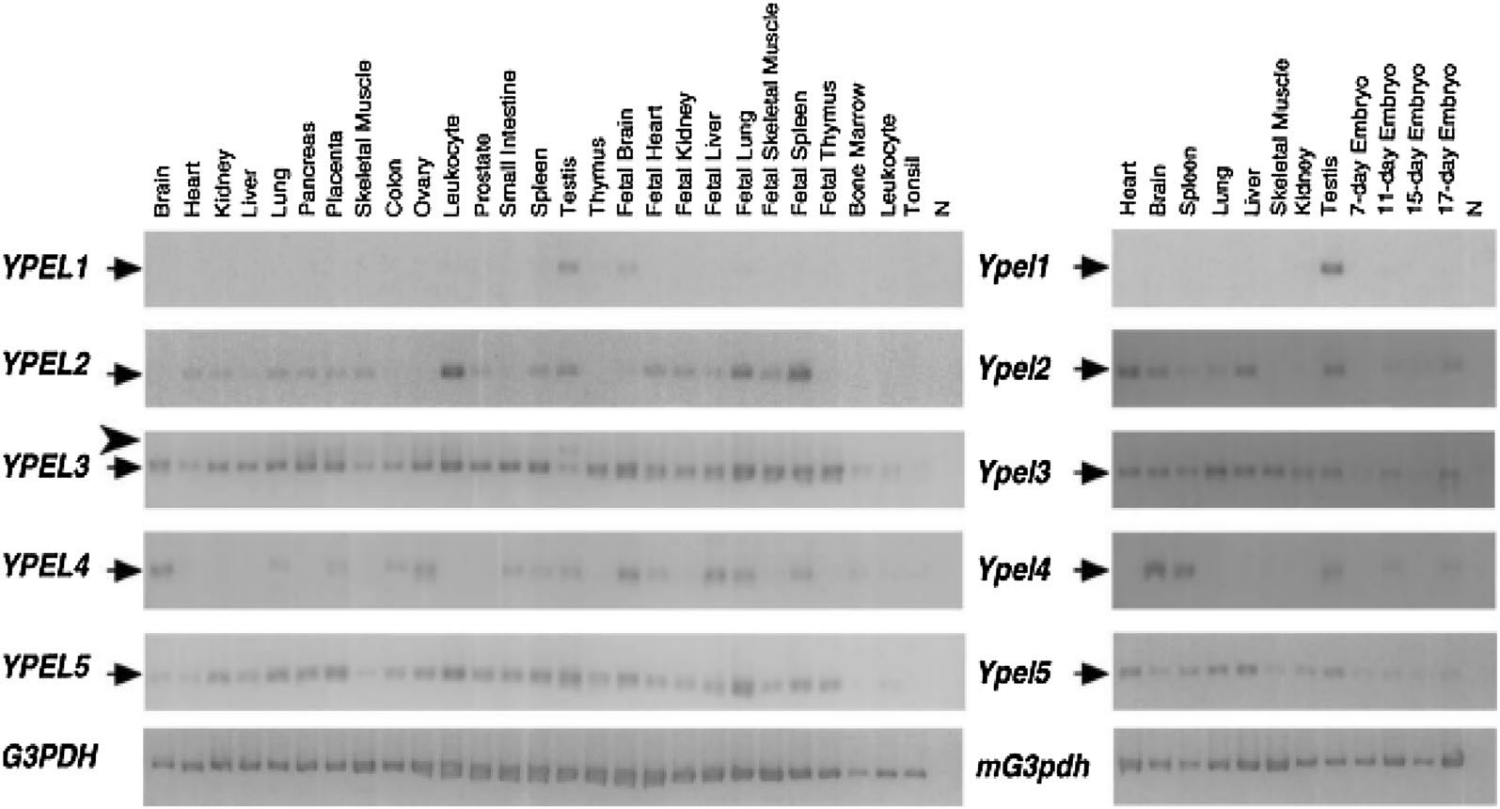
Expression profiles of human and mouse YPEL family genes in various tissues. Multiple tissues cDNA (MTC) panels were used for PCR specific to each family member with 30 cycles. PCR for YPEL2, YPEL4, Ypel2 and Ypel4 were done with 35 cycles. *N* negative control with no cDNA, G3PDH: control for cDNA amount using glyceraldehyde-3-phosphate dehydrogenase (G3PDH) [1]

Hosono et al. [1] analyzed the expression profile of the human and mouse YPEL family genes using PCR amplification of the cDNA derived from various organs and tissues. PCR primer pairs specific to each human *YPEL1*-*5* and mouse *Ypel1*-*5* genes were designed. For human *YPEL4*, the PCR primers used for cloning and expression analysis were named YPEL4.MTC.F1 and YPEL4.MTC.R1. The sequences for these primers were 5′-CCACCAAGACTTTCCGCAGCTAT-3′ and 5′-GTACTCATGCTGGTATAGACTGC-3′, respectively. YPEL4 was expressed in brain, lung, placenta, colon, ovary, small intestine, spleen, testis, fetal brain, fetal heart, fetal liver, fetal lung, fetal spleen and bone marrow [1]. Hosono et al. noted that the expression profile of *Ypel* family genes in mouse organs and tissues were almost identical to those in human. The expression profiles of human *YPEL1*-*5* and mouse *Ypel1*-*5* can be seen in Fig. 2.

## *YPEL4* locations

Although the function of YPEL4 is not entirely known, previous studies suggest that the interaction of YPEL4 and other members of the YPEL gene family have a role in the cell life cycle. Hosono et al. [1] analyzed the cellular localization of the YPEL family proteins in order to clarify their function. Cellular localization was analyzed by using antibodies specific to human YPEL1 through YPEL4 proteins (anti-YPEL1 through anti-YPEL4) and specific to human YPEL5 protein (anti-YPEL5) for indirect immunofluorescent staining of COS-7 cells. The anti-YPEL1-4 antibody stained granular structure in the nucleus and an area adjacent to the nucleus, which appears to be the centrosome, thus, Hosono et al. employed anti-gamma tubulin antibody and observed co-staining between gamma tubulin and YPEL1-4, suggesting that at least some of YPEL1-4 proteins are associated with the centrosome in interphase. Thus, subcellular localization of the YPEL proteins to centrosomes and the mitotic apparatus suggest their role in the mitosis-associated function.

## Interactions of YPEL4 with signaling molecules

Recent studies have shown that YPEL4 may be of importance based on the interactions between YPEL4 and certain biological processes.

### YPEL4 interactions with mitogen-activated protein kinase signaling molecules

The mitogen-activated protein kinases (MAPKs) are protein serine/threonine kinases that are involved in the conversion of extracellular stimuli into cellular responses [4]. All eukaryotic cells have multiple MAPK pathways which regulate several processes such as gene expression, cell proliferation, metabolism, apoptosis, differentiation, and etc. Liang et al. [5] examined the relationship between YPEL4 and the MAPK pathways and identified the major vault protein (MVP), an important component involved in the MAPK signal pathway, as a binding protein of YPEL4. Prior to their discovery, there were no reports of YPEL4-interacting factors. To identify MVP as a protein partner of YPEL4, Liang et al. searched for YPEL4-interacting proteins using YPEL4 as the bait and screened a human brain cDNA library in a yeast two-hybrid system. This interaction between YPEL4 and MVP was later confirmed through several assays including mammalian two-hybrid reporter assay, co-localization assays, co-immunoprecipitation, and GST pull-down assay. Liang et al. [5] also used *RNAi* analysis and luciferase reporter assay to demonstrate that MVP suppressed YPEL4-mediated Elk-1 activation. The vector constructs used through the assays are summarized below in Table 1.

**Table 1:** Vector construction used for the yeast two-hybrid screening and other assays by [5]

Type of assay	Final vector	Vector construction
Yeast two-hybrid screening	pGBKT7-YPEL4	Full-length cDNA of YPEL4 was ligated in frame with GAL4 DNA-binding domain of the pGBKT7 vector
Mammalian two-hybrid reporter assay	YPEL4 expression vector: pCMV-BD-YPEL4	Full-length cDNA of YPEL4 was cloned into mammalian expression plasmid pCMV-BD
	MVP expression vector: pCMV-AD-MVP	cDNA fragment of MVP was cloned into mammalian expression plasmid pCMV-AD
Co-localization assay	Vector EGFPN1 was used to construct vectors expressing YPEL4, and vector pCMV-Tag2B was used to construct a vector expressing MVP	
GST pull-down assay	pGEX-4T-1 was used to construct vectors expressing GST-YPEL4 fusion proteins	
Immunoprecipitation assay	pCMV-Tag2B and pCMV-Myc were used to construct vectors expressing YPEL4 and MVP, respectively	

### Interaction of YPEL4 with MVP

**Fig. 3:**
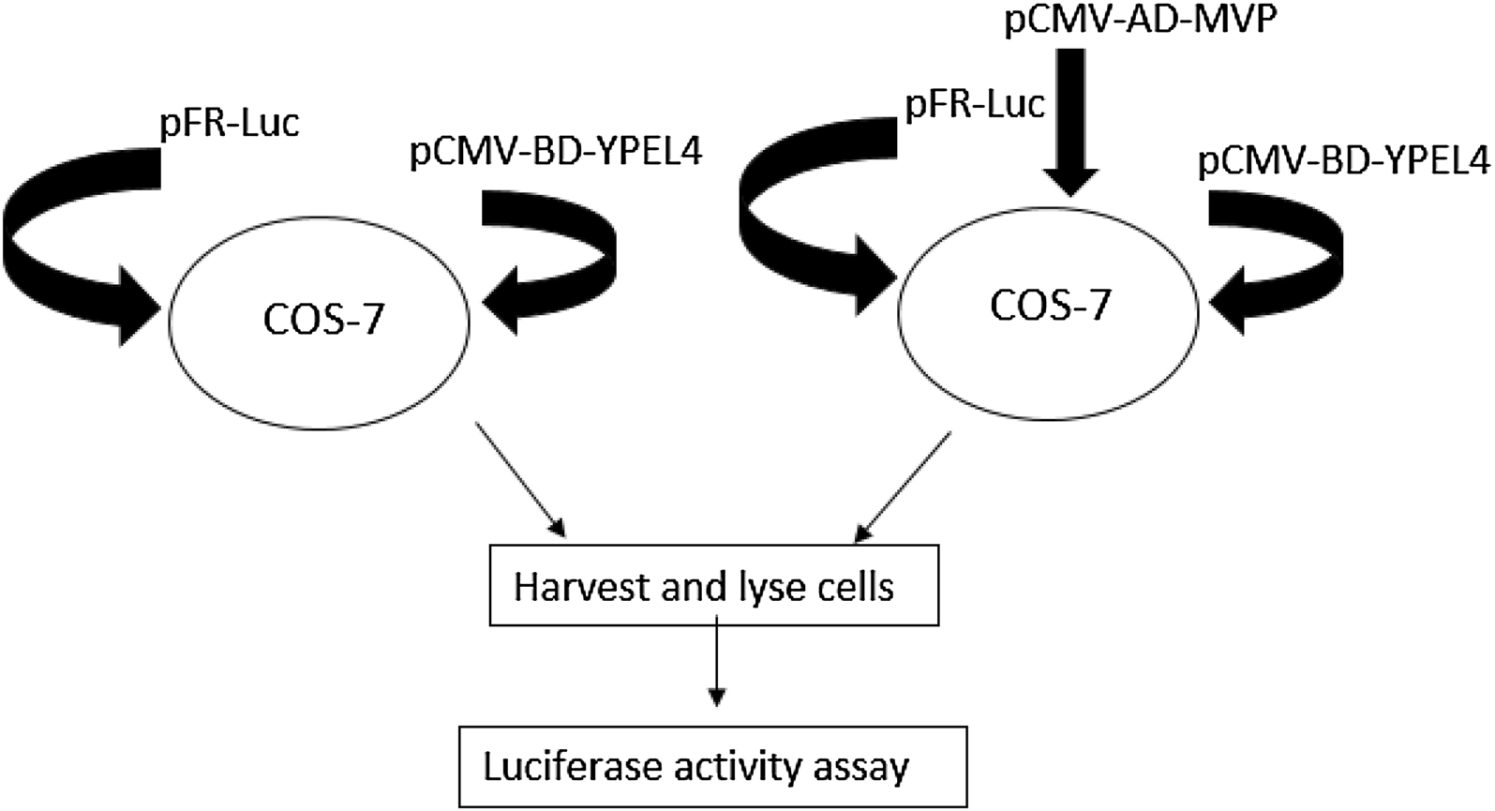
A schematic diagram of the interaction between YPEL4 and MVP. COS-7 cells were co-transfected with YPEL4 and MVP luciferase reporter plasmids. Luciferase activity was three times higher in cells co-transfected with YPEL4 and MVP than in cells transfect with YPEL4 alone (Adopted from [5])

Using COS-7 cells that were co-transfected with pCMV-BD-YPEL4, pCMV-AD-MVP, and pFR-Luc reporter plasmids, these cells were harvested and lysed for luciferase activity assays as diagrammed in Fig. 3. Cells co-transfected with pCMV-BD-YPEL4 and pFR-Luc reporter plasmid were used as the control group. Luciferase activity was measured by injection of luciferin into the cell lysate and assayed 36 h after transfection. Luciferase activities of cells transfected with the pCMV-BD-YPEL4 expression vector were three times lower than cells co-transfected with both pCMV-BD-YPEL4 and pCMV-AD-MVP. These results suggested that YPEL4 interacts with MVP in mammalian cells [5].

### Co-localization of YPEL4 and MVP

Immunofluorescence staining was done on COS-7 cells transfected with pEGFP-N1-YPEL4 and pCMV-Tag2B-MVP to confirm colocalization between YPEL4 and MVP. Cells were incubated with monoclonal anti-Flag and Cy3-conjugated phalloidin anti-mouse IgG. Cell nuclei were stained with DAPI. Co-localization of EGFP-fused YPEL4 and Flag-fused MVP was detected using a Nikon inverted fluorescence microscope. Images obtained from inverted fluorescence microscopy revealed that both EGFP-fused YPEL4 and Flag-tagged MVP were localized close to the mitotic apparatus in the mitotic phase. After overlay, co-localized signals were observed and further confirmed the presence of YPEL4 and MVP in the same region [5].

### In vitro and in vivo models of interaction of YPEL4 with MVP

Using a GST pull-down assay, Liang et al. determined whether YPEL4 could bind to MVP in vitro. GST-fused YPEL4 and GST alone were immobilized separately on glutathione-Sepharose beads, which were incubated with lysates of COS-7 cells overexpressing Flag-tagged MVP. Western blot analysis showed that Flag-MVP was pulled down by GST-YPEL4, but not by GST alone. The interaction of YPEL4 with MVP was also demonstrated by co-immunoprecipitation. COS-7 cells were co-transfected with pCMV-Tag2B-YPEL4 and pCMV-Myc-MVP. Co-immunoprecipitation was performed using anti-Flag or anti-Myc antibody. After collecting co-precipitated proteins using protein-A/G-Sepharose, the co-precipitated proteins were analyzed by Western blotting which indicated that MVP was co-precipitated with anti-FLAG antibody, and YPEL4 was co-precipitated with anti-Myc antibody. Thus, GST pull-down assay indicated that MVP binds to YPEL4 in vitro and co-immunoprecipitation showed YPEL4 interaction with MVP both in vivo and in vitro. These results suggest that endogenous proteins are present in a protein complex in cells [5].

### Role of YPEL4 interaction with MVP on regulation of Elk-1 activity in MAPK signaling pathway

The Elk-1 gene is a member of the ETS family and has been shown to play a role in regulating cell differentiation and proliferation. The protein encoded by Elk-1 gene is a nuclear target of two members of the MAPK family, extracellular signal-regulated kinase 1 and 2, ERK1 and ERK2 [6]. GST-YPEL4 proteins were expressed in *E. coli* BL21 and proteins produced were detected using Western blotting. Expression of YPEL4 was decreased by co-transfection of pSUPERRNAi-YPEL4 in HeLa cells. The over-expression of YPEL4 increased Elk-1 activity by five-fold, whereas Elk-1 luciferase activity was inhibited when pCMV-Tag2B-YPEL4 was co-transfected with pSUPERRNAi-YPEL4 [5]. The luciferase activity for pCMV-Tag2B, pCMV-Tag2B-YPEL4, pSuper, and pSuper-YPEL4 in HeLa cells was studied to demonstrate the effects of YPEL4 on transcriptional activity of Elk-1. It was shown that the luciferase activity differences between different groups were significant, and the multiple comparisons indicated that the differences existed mainly between the pCMV-Tag2B-YPEL4 group and others. The pCMV-Tag2B-YPEL4 co-transfected with pCMV-Tag2B-MVP reduced the ability of YPEL4 to activate Elk-1 substantially, thus indicating that MVP suppressed YPEL4-mediated Elk-1 activities [5].

### Influence of YPEL4 on adrenal cell proliferation

Oki et al. [7] performed several assays that were used to clarify the effects of YPEL4 on aldosterone production and cell proliferation in a human adrenocortical cell line (HAC15) and aldosterone producing adenoma (APA). It was reported that YPEL4 potentiates aldosterone production by increasing cell proliferation and found that YPEL4 expression in APA from patients was 2.4-fold higher compared with its expression in non-functioning adrenocortical adenomas (NF). Results also suggested a positive relationship between YPEL4 expression levels and tumor diameter in APA.

**Fig. 4:**
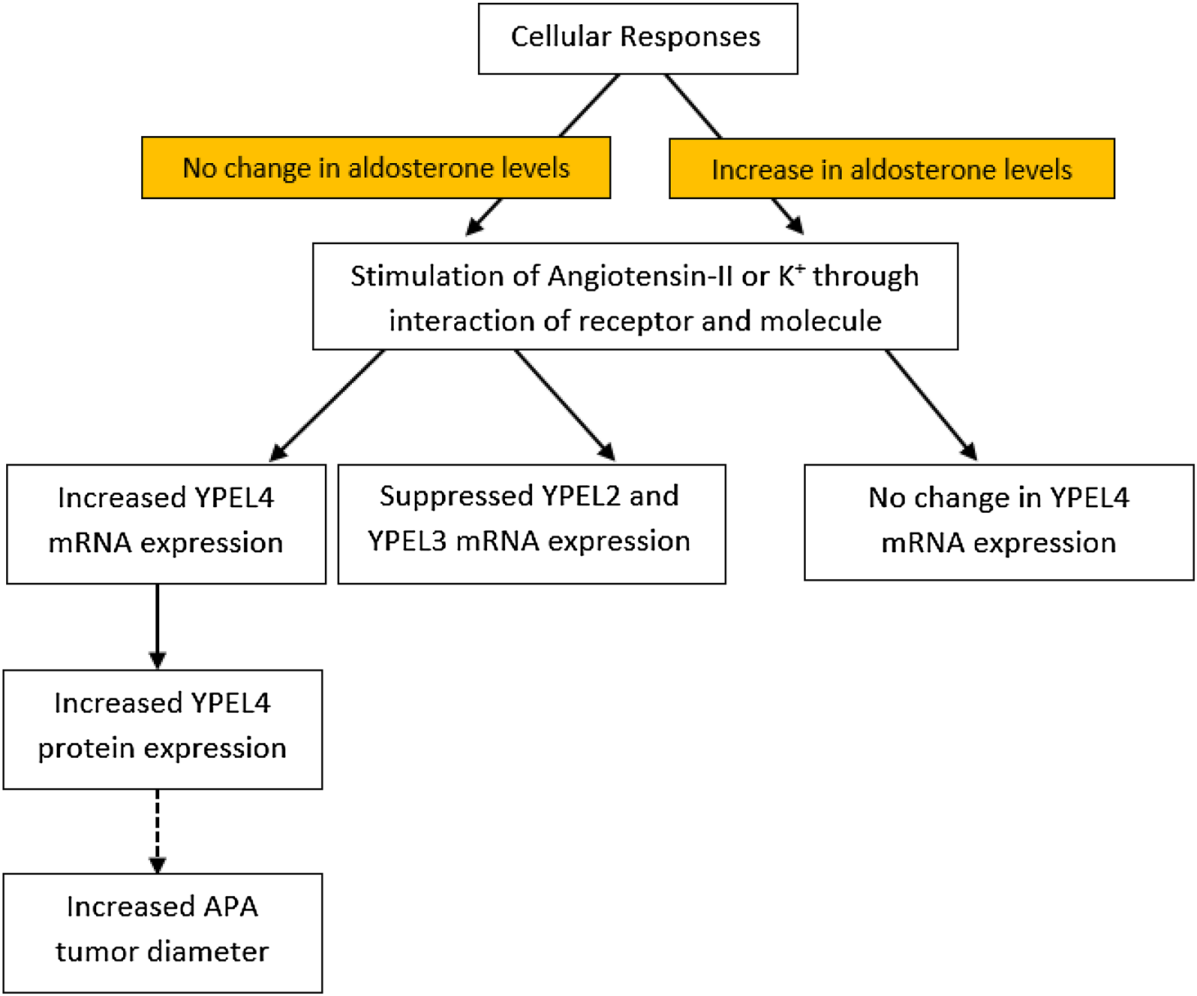
A schematic diagram illustrating the role of angiotensin-II and potassium as the molecular regulators of YPEL4 (Adopted from [7])

Interestingly, angiotensin-II (A-II) or K^+^ stimulation regulates mRNA expression levels of YPEL family members, YPEL1 through YPEL5. As shown in Fig. 4, Oki et al. found that A-II and K^+^ increased YPEL4 mRNA expression levels by 2.3 and 3.8-fold, respectively. YPEL3 mRNA levels were significantly suppressed by A-II and K^+^, 0.30-fold, and 0.60-fold, respectively and YPEL2 mRNA levels were decreased by A-II 0.57-fold [7]. Indeed, the information on the intracellular regulators of YPEL4 is limited. This may warrant further research to understand the interactions of YPEL4 with its upstream and downstream signaling molecules.

YPEL4 had an influence on several processes during this experiment such as an influence on aldosterone production, on mRNA levels of steroid synthesis enzymes, and on cell proliferation. HAC15 cells infected with YPEL4 had higher basal aldosterone levels than the controls. There were no significant differences observed between YPEL4 and controls after A-II or K^+^ stimulation. There were no differences in expression levels of mRNA for STAR, CYP11A1, HSD3B2, CYP21A2, CYP11B1 or CYP11B2 normalized to GAPDH between YPEL4 and control cells. These results indicated that YPEL4 had no effects on aldosterone production via steroid synthesis enzymes.

Oki et al. [7] determined the effect of YPEL4 in HAC15 on cell proliferation because the increase of aldosterone production was observed without a change in message for steroidogenic enzymes. The effect of YPEL4 overexpression was compared to a control using the XTT assay after 0, 24, 48, and 72 h. The number of YPEL4 and control cells were identical at the beginning of the experiment; however, the rate of proliferation of the YPEL4 cells exceeded that of controls after 24 h by a 1.31-fold increase, 1.33-fold increase after 48 h, and 1.72-fold increase after 72 h. In summary, YPEL4 stimulates human adrenal cortical cell proliferation and increases aldosterone production as a consequence.

## Involvement of YPEL4 in cancers

**Fig. 5:**
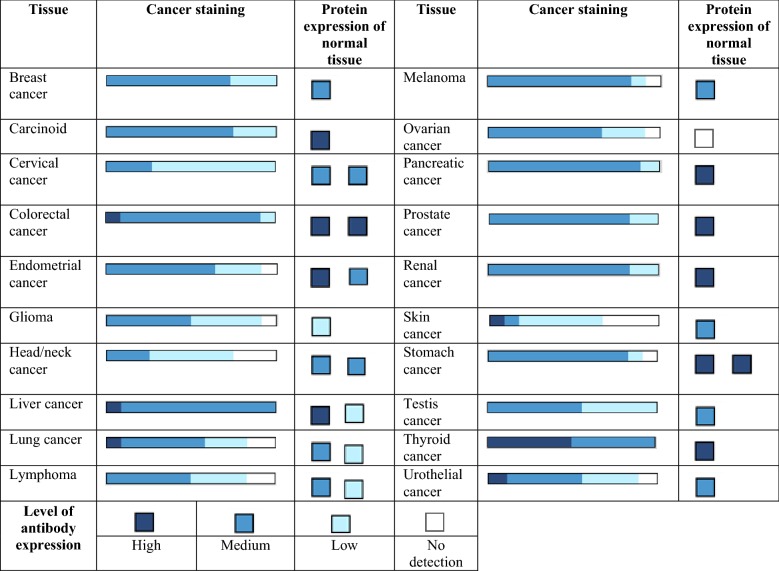
YPEL4 protein expression profiles in various cancer tissues using immunohistochemistry (The Human Protein Atlas, https://www.proteinatlas.org)

The Human Protein Atlas generates protein expression profiles using immunohistochemistry. Figure 5 shows the level of antibody staining and expression in several types of cancer including breast, carcinoid, cervical, colorectal, endometrial, lung, ovarian, skin, testis and more ([8] https://www.proteinatlas.org). When examining the level of antibody staining for protein expression, most cancer tissues exhibited weak to moderate cytoplasmic immunoreactivity. Papillary adenocarcinomas of the thyroid were strongly stained, suggesting a high level of YPEL4 protein expression ([8] https://www.proteinatlas.org). Moreover, an earlier report indicates that this molecule may play a crucial role in the development of lung cancers, particularly the non-small-cell lung cancer [9].

## Potential implications of YPEL4 in pulmonary diseases

Pulmonary diseases play a prevalent role in the death rate of developed and developing countries. However, the underlying molecular and cellular mechanisms that are involved in promoting and exacerbating these diseases are not well understood. YPEL4 has been shown to interact with several molecules such as Elk-1 and MVP within their respective pathways. Because of these interactions, it may be implied that YPEL4 has some involvement in pulmonary diseases linked to these pathways.

### Asthma

Asthma is a chronic inflammatory lung disease that affects about 25 million people [10]. This disease can be characterized by a hyperplastic increase in smooth muscle cell mass within the airway wall. This hyperplasia of the airway smooth muscle has been well documented in asthmatic patients and may be due to increased muscle proliferation.

Burgess et al. [11] looked at the dual ERK and phosphatidylinositol 3-kinase (PI3K) pathways and their regulation of airway smooth muscle cell proliferation within asthmatic patients. In non-asthmatic cells, growth is controlled by mitogens that act through the dual signaling pathways, the ERK- and PI3K-dependent pathways. In asthmatic cells, Burgess et al. found that the PI3K pathway predominates due to the upregulation of an endogenous MAPK inhibitor, MAPK phosphatase-I. This inhibitor restricts ERK signaling in asthmatic cells under mitogenic stimulation which allows the PI3K pathway to dominate. Ultimately, studies have shown that the PI3K may be an important target in asthmatic smooth muscle hyperplasia.

### Chronic obstructive pulmonary disease (COPD)

COPD is a broad term that is used to describe progressive lung diseases that include emphysema, non-reversible asthma, chronic bronchitis, and some forms of bronchiectasis. COPD is the third leading cause of death in the United States [12]. Cigarette smoking has been known to be the principle risk factor in this disease. Recent studies have investigated the role in MAPK signaling pathways that lead to changes in epithelial gene expression and cellular function. These changes may result in permanent injury and destruction to the airway and lung matrix [13].

There are several inhibitors in the MAPK signaling pathway that could be used as potential therapeutics in COPD patients. These inhibitors show selective affinity for specific kinases such as PD98059, CI-1040, and UO126 [13]. These drugs have been shown to block certain cellular behaviors associated with COPD in culture, such as production of proteases, secretion of mucus, and cell proliferation.

There are several proteins that protect against the harmful effects caused by cigarette smoking, such as oxidative stress and toxicity generated by the toxic compounds found in cigarettes. These proteins include the multidrug resistance-associated protein-1 (MRP1), P-glycoprotein (P-gp), and lung resistance-related protein (LRP). LRP is the major vault protein encoded by the major vault protein (MVP) gene. The MVP protein has been documented to interact and co-localize with YPEL4 as shown by Liang et al. [5]. van der Deen et al. [14] investigated the roles of MRP1, P-gp and LRP in COPD patients and found that there was lower MRP1 expression but normal P-gp and LRP expression in bronchial epithelium in COPD patients.

### Lung cancer

Lung cancer accounts for more deaths than from breast, colon, and prostate cancer deaths combined and approximately 14% of all new cancers are lung cancers [15]. Dingemans et al. [9] investigated the expression of the human major vault protein LRP in human lung cancer samples in comparison to normal lung tissues and found that LRP expression was significantly higher in non-small-cell lung cancer samples than small-cell lung cancer samples. Typically, all small-cell lung cancers are characteristic of low levels of LRP expression. Because LRP is a protein encoded by MVP, which interacts with YPEL4, targeting YPEL4 may affect the interaction with LRP and pose as a potential treatment for non-small- cell lung cancer where LRP expression is higher.

### Pulmonary fibrosis

Pulmonary fibrosis is a progressive fibrotic lung disease that results in tissue scarring, ultimately interfering with the ability of patients to breathe. It is difficult to determine the number of people affected by pulmonary fibrosis due to the fact that there is a large number of conditions that may cause pulmonary fibrosis [16]. Interestingly, Tatler et al. [17] identified a region of the ITGB6 promoter that is responsible for transcription repression and demonstrated that Elk1 can act to repress the *ITGB6* gene. It was also shown that an Elk1 deficiency enhanced gene expression and exacerbated induced pulmonary fibrosis in mice. It was also showed that pulmonary fibrosis patients were characterized by reduced expression of Elk1 which was associated with reduced Elk1 binding to the *ITGB6* promoter. Presumably, YPEL4 and Elk1 may be possible targets in treating pulmonary fibrosis [5].

## Inhibition of YPEL4 pathway and the potential clinical relevance

Currently, there are no YPEL4 inhibitors available. Noticeably, the PI3 K pathway plays an important role as it pre-dominates over the non-asthmatic dual pathway system between PI3K and MAPK signaling pathways. Naturally, MVP acts to inhibit the ability of YPEL4 to activate the transcription factor Elk-1 in the MAPK signaling pathway. It is possible that a specific inhibitor of MVP in terms of its interaction with YPEL4 would allow the MAPK pathway to simultaneously work with the PI3K pathway, suggesting that a specific MVP inhibitor would serve as a therapeutic target in asthma by promoting the activity of YPEL4.

Oki et al. illustrated the role YPEL4 played in aldosterone-producing adenomas. They showed that YPEL4 is able to potentiate the production of aldosterone by increasing cell proliferation, as well as the positive correlation between YPEL4 expression and tumor diameter. This suggests that YPEL4 may be a possible therapeutic target for cancers as its function is thought to be involved with cell cycle processes due to its subcellular localization. This relationship of YPEL4 and molecular regulators that result in the positive correlation between YPEL4 and tumor diameter (Fig. 4).

## Conclusion

YPEL4 is a major member of the YPEL gene family and highly expressed in lung, heart, and other important tissues [1]. The functions of YPEL4 are poorly understood; however, studies have indicated its potential essential roles in numerous cellular responses.

**Fig. 6:**
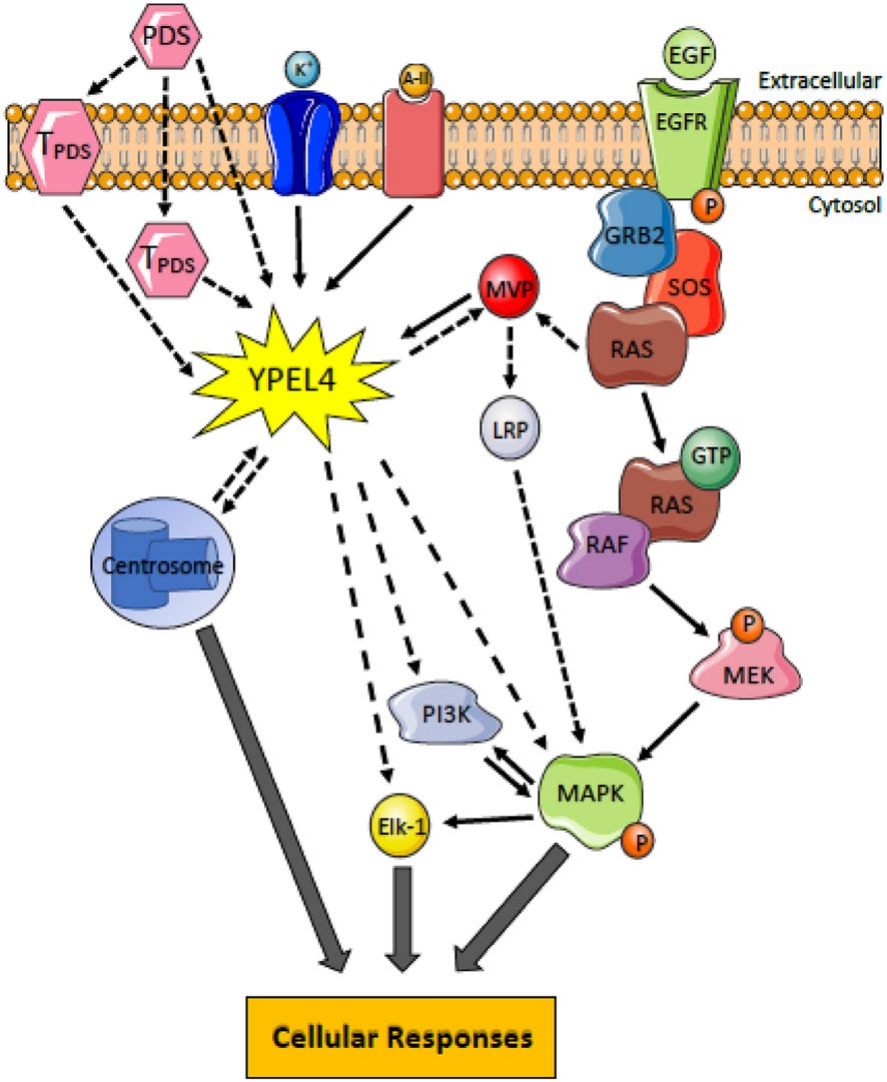
A schematic diagram illustrating the role of YPEL4 in several cellular responses. From the top, epidermal growth factor (EGF) binds to the EGF receptor (EGFR) in the cell membrane, starting the signal transduction cascade. Downstream, phosphate (P) signaling activates MAPK. MAPK then phosphorylates Elk-1, a transcription factor that regulates gene expression of ITGB6. Increased levels angiotensin-II and potassium regulate YPEL4 expression and involvement in other pathways. PDS, pulmonary disease stimuli, may lead to the overexpression or downregulation of YPEL4 through their membrane and/or intracellular target (T_PDS_), implying its involvement in pulmonary diseases. The co-localization of YPEL4 and centrosome suggest YPEL involvement in the cell division process. (Dashed arrows: indirect interactions, solid arrows: direct interactions)

As diagrammed in Fig. 6, YPEL4 is localized to the nuclei and nucleoli during interphase, suggesting its roles in mitosis-associated cellular responses [1]. By interacting with MVP, YPEL4 is involved in Elk-1 activities and functions [5]. Evidently, YPEL4 plays a significant role in the regulation of the MAPK transduction pathway. The signaling molecules described herein are crucial for the development of various hyperproliferative diseases. In support, functional studies reveal that YPEL4 mediates the cell cycle and proliferation [7]. Thus, YPEL4 may be involved in the development of several types of cancer including lung cancer ([9]; The Human Protein Atlas, https://www.proteinatlas.org). The function of YPEL4 is not fully understood, thus, further research may propel to better complete our knowledge on YPEL4 and to potentially develop therapeutic targets.

YPEL4 is the part of a complex network of pathways involved in the development of many pulmonary diseases. Studies suggest that pulmonary diseases share their molecular processes with cancers. YPEL4 may play an important role in asthma and other pulmonary diseases; thus, a series of new and meticulous studies aimed at determining the functional importance of YPEL4 and underlying signaling mechanisms in asthma and other pulmonary diseases have a very significant impact in the field.

## Abbreviations

A-II: angiotensin II
APA: aldosterone-producing adenoma
bp: base pairs
DDBJ: DNA Data Bank of Japan
EMBL: European Molecular Biology Laboratory
ERK: extracellular signal-regulated kinase
LRP: lung resistance-related protein
MAPK: mitogen-activated protein kinase
MRP1: multidrug resistance-associated protein-1
MVP: major vault protein
NF: non-functioning adrenocortical adenomas
ORF: open reading frame
P-gp: P-Glycoprotein
PI3K: phosphatidylinositol 3-kinase
PTER: phosphotriesterase-related gene
YPEL4: Yippee-like-4
